# Small-Pox Cases and Their Management during the Recent Outbreak in Buffalo, with Some Remarks on Vaccination

**Published:** 1888-10

**Authors:** Edward Clark

**Affiliations:** City Health Physician; 271 Franklin Street


Buffalo Medical and Surgical Journal

VOL. XXVIII. OCTOBER, 1888. No. 3

### Original Communications.

### SMALL-POX CASES AND THEIR MANAGEMENT DURING THE RECENT OUTBREAK IN BUFFALO, WITH SOME REMARKS ON VACCINATION.*

By EDWARD CLARK, M. D., City Health Physician.

We are informed by the authentic records of historians that variola, or, as it is commonly called, small-pox, had its origin away back in the earliest ages of the human family, among the people inhabiting the different countries of Eastern Asia. From thence this hideous and loathsome disease was carried to continental Europe, and from there to the American continent, following along the line of progress "pursued by the advancing centers of traffic and population."

Perhaps the first writer to whom we are indebted for an account of the description and ravages of small-pox during the early centuries of the Christian era, was an Egyptian physician named Ahron, who flourished in the sixth century. His views concerning the malady were graphically set forth in the tenth century by Rhazes, of Bagdad, in a very able monograph devoted to the consideration of small-pox and measles. By reference to a translation of this work made by Greenhill for the London Pathological Society in 1848, it is very evident that Rhazes has given us a remarkably accurate and life-like portraiture of the clinical and anatomical characteristics of the dread disease:

"After these, dates the remarkable political and social changes in Europe, which are to be attributed either directly or remotely

* Read before the Buffalo Medical and Surgical Association.

to the crusades, contributed largely to the opportunities for the spread of the disease, and to the occurrence later of those decimating epidemics which became veritable scourges. In the last century the resulting mortality in some of the countries of Europe was often equal to the entire population of one of their largest cities. If a modern traveler could find himself transported to the streets of the City of London as they appeared in the early part of the present century, it is probable that no peculiarities of architecture, dress or behavior would be to him so strikingly conspicuous as the great number of pock-marked visages he would encounter at every turn."

It is, perhaps, as rare a sight now to see a pock-marked face in a civilized country, as it was to see a face which was not pock-marked before the illustrious Sir Edward Jenner discovered the prophylactic power of vaccination toward the close of the eighteenth century.

In spite of all cavil, ignorant prejudice and superstition, medical science to-day, and for all time to come, will point to this wonderful discovery as one of the greatest achievements the world has ever seen.

Different parts of our own country have, from time to time, suffered from visitations of small-pox, which, in some instances, have made sad havoc among the inhabitants of the places attacked. Buffalo has suffered from several outbreaks of small-pox, perhaps the most severe of which occurred in 1872 and 1873. During the past two months a number of persons in this city have been attacked with small-pox, the first victim being a well-known colored man, "Prof. John Jackson, the champion whitewasher," who, for many years, was a familiar figure about town. I was first called to see him July 7th by Dr. G. W. York, who suspected the disease to be small-pox on the day before I saw the case. There was no possibility of mistaking the disease for anything else at the time of my first visit. The house was immediately placarded and quarantined, and all the other inmates vaccinated. Jackson was not removed to the Quarantine Hospital on account of his feeble, debilitated condition. He had for a long time been suffering from rheumatism and partial hemiplegia, and, in fact, was in an almost dying condition at the time of his attack. He died about seventeen hours after I was first called to him, and was buried five hours after death. All the bedding and hangings in the room in which he died were immediately burned, and the house thoroughly fumigated. Mrs. J. used the rear rooms of the house for laundry purposes, and at the time of her husband's decease she had a large quantity of laundried clothing on hand ready to be delivered. This was all thoroughly soaked in a strong bichloride solution, and the woodwork and floors thoroughly scrubbed with the same. The walls of the house were then whitened with a mixture containing bichloride, and the inmates of the house were closely confined for ten days. No new cases have since developed in that neighborhood.

About ten days later, nine cases were found within a day or two among the Polish people on the east side. The density of the population, the crowded condition of the houses, the number of unsewered streets, and the large proportion of unvaccinated children in this section of the city, would naturally lead anyone to believe that this would be a most prolific field for the growth and development of small-pox. These patients were all removed to the hospital as soon as discovered, and rigid quarantine regulations were enforced at each house where the disease had made its appearance. Upon careful inquiry I learned that some of the children who were afflicted with the disease had been in attendance at the parish school after the eruption had begun to show itself. This school is connected with the St. Stanislaus Polish church, on Peckham street, and has an average daily attendance, so I am informed, of nearly 1,800 children, not more than fifty per cent. of whom had ever been vaccinated.

With a knowledge of these facts in my possession, I was forced to the conclusion that the only way to prevent the disease becoming epidemic in that locality was to enforce general vaccination throughout the whole Polish community. Accordingly, with the consent and approval of the Board of Health, I opened a Bureau of Vaccination right in the heart of the Polish district, and calling to my aid the entire medical corps of the Health Department—sixteen physicians in all—proceeded with a thorough house-to-house vaccination. I wish to say, at this time, that the members of the medical staff of the Board of Health are entitled to great credit for the promptness with which they responded in this emergency. Many of them gave up their private business entirely, and worked as many as twelve hours a day in helping to stamp out the scourge. The work was kept up until nearly all of the inhabitants living in that portion of the city bounded by William, Smith, Sycamore, and Rother streets were vaccinated—about 16,000 in all. The Polish people, with very few exceptions, showed a commendable willingness to assist the physicians in their efforts to enforce vaccination; hundreds of them, with their little ones, would come to the office daily to be vaccinated. The wisdom of this general vaccination is evidenced by the fact that since the first case was discovered among the Poles, only two or three persons have developed small-pox in that community, and investigation showed that all the persons in that section of the city who had the disease, were either relatives or had very intimate associations with each other. One of these patients, a child three and one-half years of age, was dead when I first saw it. This was the case where the attending physician certified that the cause of death was "purpura hemorrhagica." The funeral was in progress, and the casket containing the remains of the child was just being brought out of the church, when I arrived on the ground and demanded the body for examination. This was about nine hours after the death of the child. An inspection of the body at once revealed the fact that the child had hemorrhagic small-pox well advanced. The father of the child stated as a reason for so hurried a funeral, that "the child smelled so badly and was peeling so much that he was afraid it would fall to pieces."

About the middle of August, four or five cases were discovered in widely remote parts of the city; only one, however, on the west side, which was on Fifteenth street. On the east side, cases were found on Cedar street, one on Eagle street, and a very mild case on Watson street. These patients were all removed to the hospital, and the usual quarantine regulations established. Eight or ten days later, two cases occurred at the corner of Monroe and Beckwith streets, and four days later, two more children in the Watson-street house were taken ill with the disease, and three patients were removed from the Cedar-street house with variola; one of them a man sixty-five years of age. About this time a case was discovered in a young woman aged twenty-three, living at 154 Van Rensselaer street, and in a boy aged seventeen, living at No. 30 Box avenue. A few days later a woman about twenty-six years of age, living at 66 Peach street, was taken ill with small-pox. She was a relative of the family living on Cedar street, where four cases had already occurred. Then three more cases developed in the Best-street house. The next case was a young man twenty-six years old, living at 121 Carroll street, and about the same time a child three years of age, living on Lombard street, was taken sick with the disease.

The following table shows the total number of cases which have occurred in the city since July 7th up to date, together with the age of patient, type of disease, result, etc.:

| No. | Name. | Age. | Type. | Date. | Result. |
|---|---|---|---|---|---|
| 1 | John J.* . . | 65 | Semi-confluent . . | July 8 | Died . . . . . . . . . . . |
| 2 | John R. . . | 45 | Varioloid . . . . . | " 16 | Recovered . . . . . . . . . |
| 3 | Joseph R. . . | 10 | Confluent . . . . . | " 16 | Recovered . . . . . . . . . |
| 4 | Rosa R. . . | 7 | Discreet . . . . . | " 16 | Recovered . . . . . . . . . |
| 5 | Mary R. . . | 3 | Discreet . . . . . | " 16 | Recovered . . . . . . . . . |
| 6 | Joseph S . . | 9 | Confluent . . . . . | " 16 | Recovered . . . . . . . . . |
| 7 | Rosa F. . . . | 7 | Confluent . . . . . | " 16 | Recovered . . . . . . . . . |
| 8 | Mary K. . . | 3 | Confluent . . . . . | " 17 | Recovered . . . . . . . . . |
| 9 | James G. . . | 13 | Discreet . . . . . | " 27 | Recovered . . . . . . . . . |
| 10 | Joanna H. . | 5 | Confluent . . . . . | " 28 | Recovered . . . . . . . . . |
| 11 | Katie H. . . | 3 | Hemorrhagic . . . | " 28 | Died twenty-ninth of July . . |
| 12 | Mrs. M. S. . | 34 | Confluent, 2d attack | " 30 | Recovered . . . . . . . . . |
| 13 | Mary G. . . | 6 | Confluent . . . . . | " 31 | Recovered . . . . . . . . . |
| 14 | David B. . . | 47 | Confluent . . . . . | Aug. 17 | Recovered . . . . . . . . . |
| 15 | Georgie K. . | 6 | Confluent . . . . . | " 17 | Died August 28th . . . . . |
| 16 | Clara E. . . | 15 | Confluent . . . . . | " 18 | Recovered . . . . . . . . . |
| 17 | Mary P. . . | 3 | Discreet . . . . . | " 18 | Recovered . . . . . . . . . |
| 18 | Grace K.* . | 9 | Hemorrhagic . . . | " 20 | Died at home . . . . . . . . |
| 19 | Georgie B. . | 3 | Confluent . . . . . | " 22 | Died at hospital . . . . . . . |
| 20 | Rosa L. . . | 4 | Confluent . . . . . | " 27 | Died September 7th . . . . . |
| 21 | Georgie L. . | 2½ | Confluent . . . . . | " 27 | Recovered . . . . . . . . . |

| No. | Name. | Age. | Type. | Date. | Result. |
|---|---|---|---|---|---|
| 22 | Hannah P. . | 10 | Discreet . . . . . | Aug. 28 | Recovered . . . . . . . . . . |
| 23 | George P. . . | 6 | Discreet . . . . . | " 28 | Recovered . . . . . . . . . . |
| 24 | Katharine K. | 32 | Semi-confluent . . | " 30 | Recovered . . . . . . . . . . |
| 25 | Martha S. . . | 18 | Varioloid . . . . . | " 30 | Recovered . . . . . . . . . . |
| 26 | Christian S. | 65 | Discreet . . . . . | " 30 | Recovered . . . . . . . . . . |
| 27 | Mary Q. . . | 23 | Semi-confluent . . | " 30 | Recovered . . . . . . . . . . |
| 28 | Edmond H. | 17 | Confluent . . . . . | " 31 | Died . . . . . . . . . . . . . |
| 29 | Baby R.* . . | 3½ | Hemorrhagic . . . | . . . | Died at home. } Both dead when |
| 30 | Baby N.* . . | 6 mos. | Discreet . . . . . | . . . | Died at home. } I first saw them |
| 31 | Mary K.* . | 10 | Confluent . . . . . | . . . | Recovered at home . . . . . |
| 32 | Baby E. . . | 7 mos. | Confluent . . . . . | . | Taken sick in hosp., died there. |
| 33 | Carrie S. . . | 30 | Discreet . . . . . | Sept. 1 | Recovered . . . . . . . . . . |
| 34 | Gertie B.* . | 7 | Hemorrhagic . . . | " 3 | Died at home . . . . . . . . |
| 35 | John B. . . . | 6 | Confluent . . . . . | " 3 | Died at hospital . . . . . . . |
| 36 | Rosie B. . . | 8 | Confluent . . . . . | " 3 | Died at hospital . . . . . . . |
| 37 | Theodore N. | 4 | Confluent . . . . . | " 4 | Recovered . . . . . . . . . . |
| 38 | Jessie S. . . | 26 | Varioloid . . . . . | " 4 | Recovered . . . . . . . . . . |
| 39 | John N.* . . | 7 | Discreet . . . . . | " 6 | Recovered at home . . . . . |
| 40 | George B. . | 7 | Confluent . . . . . | " 14 | Recovered at hospital . . . . |
| 41 | Mrs. S.* . . | . . | Varioloid . . . . | " 15 | Recovered at home . . . . . |

From the above table it will be seen that there have been, in all, forty-one cases. Of this number, thirty-three were admitted to the Quarantine Hospital, eight of whom have died, a mortality of twenty-four and one-quarter per cent. Of the eight patients who were not removed to the hospital, five died, a mortality of sixty-two and one-half per cent.

Twenty-four of the patients had confluent small-pox, four ot whom developed the hemorrhagic type. Three cases were semi-confluent, and ten were of the discreet variety. There were four cases of mild varioloid. The youngest patients were two nursing babies, one seven months of age, who contracted the disease in the hospital, and died; the other, six months of age, died at home; while the oldest patients were two men, aged respectively sixty-five years, one of whom died. Twenty-five of the patients were under ten years of age, four between ten and twenty, two between twenty and thirty, two between thirty and forty, two between forty and fifty, and two between sixty and seventy.* All of the patients that died were under nine years of age, except two, one of whom, "Prof." J., was sixty-five, and the other a boy aged seventeen. Of the persons attacked with the disease, there were twenty-four females and seventeen males. Of those in whom the disease proved fatal, there were eight females and five males. All the hemorrhagic cases proved fatal.

In comparing the rate of mortality in the hospital with that outside, it should be borne in mind that of the patients who were not removed from their homes, three had the hemorrhagic orm of the disease. One, "Prof." J., was a very feeble, debilitated person, who would in all probability have succumbed to a severe attack of any malady whatever. Another one was a baby six months of age, which died before the eruption was very much manifest.

When the first cases of small-pox were sent to the Quarantine Hospital, I appointed Dr. C. B. Le Van, resident physician of the institution. I believe this is the first appointment of the kind ever made. Dr. Le Van is a young man of excellent ability, and he has given the best of satisfaction in a very trying position. The patients have received the benefit of his constant care and devotion, together with almost daily visits from myself. In the editorial language of my esteemed friend and associate, the late Dr. F. R. Campbell, "The wisdom of making such an appointment is shown by the result: a large proportion of recoveries, the amount of disfiguration from the disease which has been greatly reduced, and the dread which patients have hitherto had of going to a suburban hospital with no physician in constant attendance, has been in a great measure removed."

A brief description of the plan of treatment inaugurated and carried out at the hospital may not be inappropriate. The cases have all been treated expectantly. The principal drugs used are quiniæ sulph., antifebrin, digitalis, aromatic spirits of ammonia, potassium bromide, salicylate of bismuth, tinct. opii camph., tinct. catechu, morphia sulph., tinct. chincona co., tinct. ferri chlor., and alcoholic stimulants. Antifebrin is the drug relied on to reduce the pyrexia in both the primary and secondary fevers; quinine is not given for this purpose, but is used chiefly with reference to its tonic and antiseptic properties. Bromide of potassium, combined with a small quantity of chloral hydrate, is used for the delirium, which is not an unusual accompaniment of the primary fever, and sometimes also of the secondary type.

In those cases where great depression occurred, associated with symptoms of collapse, digitalis, aromatic spirits of ammonia and alcoholic stimulants were quite freely used. During the stage of dessiccation and later on, a great many of the patients were attacked with an obstinate form of diarrhœa, which, for a time, seemed to resist the remedies used to check it. In most cases, however, it finally yielded to a persistent use of the salicylate of bismuth with tinct. opii. camph. and tinct. catechu. In some of the older patients, morphia sulphate was also brought into requisition, and was followed by very satisfactory results. Bitartrate of potash has been highly spoken of by many as having a special efficacy in the therapeusis of variola. We gave this drug a very fair trial at the Quarantine Hospital, but, so far as could be ascertained, without any marked effect on the progress of the disease.

In the way of local treatment, the following plan has given very satisfactory results in mitigating the irritation and pain, and preventing, to a great extent, the pitting and marking of the exposed parts of the body. The vesicles on the face and hands, and the larger vesicles wherever found, are opened freely with a very small, well-sharpened tenotomy knife, and their contents are then thoroughly extracted by means of a small soft sponge rung out of a warm solution of boracic acid, one drachm to a pint of water. The face, hands and neck are frequently thereafter bathed in the same solution. This sponging and bathing process is kept up until the close of the pustular stage, from which time the surface of the body is kept thoroughly anointed with boracic acid ointment, fifteen grains to one ounce of cosmoline, until desquamation is pretty thoroughly advanced. I also tried, at the suggestion of Dr. B. H. Daggett, on the face and hands of a few patients, a paste made of oxide of zinc and glycerine. I am not so well pleased with its action, however, as I am with that of the boracic acid ointment.

At the request of one of our most experienced practitioners, I used, in the early stages of the disease, a mild bichloride solution as a wash for the hands and face, in order to see what effect, if any, it would have in aborting or allaying the progress of the malady. After giving it a fair trial, I am free to say, that so far as could be observed, its results were entirely negative.

A remedy which is regarded by some physicians as a specific for small-pox, is the root of *sarracenia purpurea*, or, as it is commonly called, pitcher plant and huntsman's or Indian's cap. An infusion of this drug has been freely used both as a curative and preventive agent with (it is claimed) very satisfactory results. I have had no experience with it, but if I could procure a reliable sample of the drug, I should be induced to give it a trial.

In concluding this article, I wish to briefly allude to the only rational treatment of variola, viz., the preventive treatment, or vaccination. A large majority of the people composing the laity undoubtedly labor under the popular delusion that a successful vaccination in infancy insures a life-long immunity from small-pox. Possibly it may be true that a few persons are protected for a life-time by such vaccination, but they constitute a very small minority. Many also believe (to use a popular expression) "that it runs out every seven years." There are others also who think that so long as their arms can show a well-marked scar, they are safe. I am sorry to say that among this number there are physicians, as recent experiences prove. In the face of so much error and conflict of opinion, is it not the duty of the medical profession to enlighten the community at large concerning this important subject? An eminent medical writer has recently said of this matter, that "the loftiest end to be reached by the physicians of our day with respect to variola, is its complete removal from all civilized countries, and, indeed, from the face of the earth, by the universal practice of vaccination and re-vaccination." A large contingent of our foreign population do not believe in the prophylactic power of vaccination, and during the recent outbreak of small-pox in this city, it became necessary to call out the armed forces of the law to coerce some of them into submission. Many families were found in which several members had passed the age of fourteen without ever having been vaccinated. In view of these alarming facts, is it not high time for the constituted authorities to interpose, and, by compelling such persons to submit to a harmless process, protect not only themselves but the welfare of the community in which they live? My individual opinion is, that our State Legislature should, in the near future, as a defense to common humanity, enact laws, under the provisions of which "every unvaccinated man, woman and child would be regarded as an enemy of the human race and treated accordingly." I hope ere long to see a stringent law passed compelling the parents and guardians of children to have them vaccinated by the time they are one year of age, and every three years thereafter, until they attain their majority. If this were done, after a few years such a thing as an outbreak of small-pox would be an unknown occurrence in our commonwealth. I am convinced that the only safe rule to follow, as regards vaccination, is to have it done as often as there is any danger from small-pox outbreaks, even if it is twice a year, and all persons should be vaccinated every three or four years, whether there are cases of small-pox prevailing or not. If the first vaccination does not work, it should be tried again and again for at least a half dozen times. I recently vaccinated an adult member of my own family eight times before it worked successfully. A man sixty-five years of age recently entered the hospital with small-pox, who was successfully vaccinated two years ago. Many patients, after being vaccinated, think that if the arm gets sore at all it is evidence of a successful vaccination. I think that physicians should try and see for themselves whether or not the persons whom they vaccinate develop a true vaccine pustule, and no case should be pronounced successful unless the sore passes through the characteristic stages of vaccinia. I believe that in primary vaccinations a single application is not sufficient, but that two or three pustules should be produced at least. I believe the custom of making a single sore on the arm affords an explanation why so many children who have been recently vaccinated contract variola. A single sore is not sufficient protection. It seems an almost universal belief that one attack of small-pox is a sure preventive against a future occurrence of the disease in the same patient. This is not so, and such persons should also be vaccinated. A woman thirty-four years of age was recently admitted to the hospital suffering from confluent small-pox, who had a similar attack when she was six years of age, for which she was sent to a German hospital.

A word or two in conclusion regarding vaccine virus. The arguments in favor of the use of good bovine virus are so many and so strong at the present time, that very few intelligent physicians can be found who would use any other kind. The competition, however, between different propagators has grown so active, and the demand for virus at times is so great, that perhaps we cannot always rely on the article furnished us. I am of the opinion that this difficulty could be remedied by having all the virus used by Boards of Health prepared under the supervision of the State Board of Health, or by a commission appointed for the purpose. The State should bear the expense, and in this way the health authorities could always get a reliable article at actual cost.

271 Franklin Street, September 18, 1888.

